# The impact of preoperative biliary drainage on bile colonization of patients undergoing pancreaticoduodenectomy

**DOI:** 10.1080/07853890.2025.2540024

**Published:** 2025-07-31

**Authors:** Yifei Yang, Yichen Duan, Chengxiang Su, Jianjie Sheng, Linxi Zhu, Yu Xie, Haobai Liu, Neng Tang, Yudong Qiu, Chenglin Lu, Chuang Chen, Liang Mao, Xu Fu

**Affiliations:** ^a^ Department of Pancreatic and Metabolic Surgery, Nanjing Drum Tower Hospital, the Affiliated Hospital of Medical School, Nanjing University, Nanjing, China; ^b^ Department of Critical Care Medicine, Jinling Hospital, the Affiliated Hospital of Medical School, Nanjing University, Nanjing, China; ^c^ Department of Pancreatic and Metabolic Surgery, Nanjing Drum Tower Hospital Clinical College of Nanjing University of Chinese Medicine, Nanjing, China; ^d^ Cardiovascular Medical Center, Nanjing Drum Tower Hospital, the Affiliated Hospital of Medical School, Nanjing University, Nanjing, China; ^e^ Department of Hepatopancreatobiliary Surgery, The Affiliated Huai’ an Hospital of Xuzhou Medical University, Huai, China’an

**Keywords:** Pancreaticoduodenectomy, surgical site infection, preoperative biliary drainage, bile contamination

## Abstract

**Background:**

Preoperative biliary drainage (PBD) may be performed for jaundiced patients with periampullary tumors. This study aimed to evaluate the impact of PBD on biliary microbiome and perioperative complications in patients undergoing pancreaticoduodenectomy (PD).

**Methods:**

This retrospective study enrolled 323 patients who underwent PD between March 2018 and March 2024. Intraoperative bile specimens were obtained for microbiological analysis of species identification and antimicrobial resistance patterns..

**Results:**

PBD was performed in 191 (59.1%) of the 323 patients. Organ/space surgical site infection (SSI) (51.8% vs 37.9%, *p* < 0.001) and bacterial colonization of bile (90.6% vs 28.0%, *p* < 0.001) were significantly more frequent in patients with PBD. PBD was identified as an independent risk factor of organ/space SSI (OR = 1.837, 95% CI: 1.158–2.916, *p* = 0.010) and associated with increased contamination with polymicrobial mixed flora (47.1% vs 4.5%, *p* < 0.001), *K. pneumoniae* (23.6% vs 0.8%, *p* < 0.001), *E. faecalis* (14.1% vs 1.5%, *p* < 0.001), *E. faecium* (6.8% vs 0.8%, *p* = 0.009). This shift corresponded to higher resistance to piperacillin–tazobactam (23.1% vs 0.0%, *p* = 0.038), cefoperazone–sulbactam (25.3% vs 0.0%, *p* = 0.021), ciprofloxacin (36.1% vs 6.3%, *p* = 0.006), and levofloxacin (47.4% vs 4.8%, *p* < 0.001). Patients with positive bile culture had a significantly higher occurrence of organ/space SSI than the negative group (53.3% vs 32.7%, *p* < 0.001). *K. pneumoniae* was identified as an independent risk factor for organ/space SSI (OR = 2.636, 95% CI: 1.353–5.137, *p* = 0.004).

**Conclusions:**

There were fundamental differences in the bile microbiome profile and antibiotic resistance of patients with/without PBD. These findings suggest that adjusting perioperative antibiotic regimens based on biliary culture may be warranted.

## Introduction

Pancreaticoduodenectomy (PD) remains the primary surgical procedure for the treatment of several benign and malignant diseases localized in the periampullary region [1,2]. Nevertheless, postoperative morbidity, including clinically relevant postoperative pancreatic fistula (CR-POPF) and organ/space surgical site infection (SSI), still ranges between 30 and 50% [3,4].

The optimal management strategy for patients with preoperative jaundice undergoing PD-immediate surgery versus preoperative biliary drainage (PBD)-remains debated. While, PBD was historically advocated to restore hepatic function and coagulopathy [5,6], contemporary evidence challenges this paradigm and shows significantly higher postoperative morbidity among patients with PBD [3,7,8]. Meanwhile, our prior studies identified that PBD was one of the risk factors of postoperative infectious complications after PD [9,10]. This apparent discrepancy in findings may reflect the evolving nature of the evidence, potentially influenced by differences in study design, patient selection criteria (e.g. severity of jaundice, presence of cholangitis), technical aspects of drainage procedures (e.g. endoscopic vs. percutaneous), or perioperative management practices (e.g. antibiotic regimens) over time.

A significant concern raised by PBD is that duodenal juice refluxes into the normally sterile biliary tree and causes bacterobilia [11]. Several studies proposed that microorganisms colonized in bile caused by PBD could develop antibiotic resistance, contributing to infectious complications after PD [12,13]. Despite these evidences and concerns, PBD remains a common clinical practice, particularly in cases with severe hyperbilirubinemia. Critically, alterations in biliary microbiome profiles (including diversity, dominant taxa distribution, and polymicrobial colonization) along with resistance patterns to prophylactic antibiotics and their clinical correlations with postoperative complications (particularly organ/space SSI) in patients with/without PBD remain incompletely characterized.

Building on these, the current study aimed to characterize biliary microbiome alterations (such as composition and poly microbial colonization) in periampullary cancer patients stratified by PBD exposure, asses the resistance rate among bile isolates to classic prophylactic antibiotics and evaluate the potential associations between microbiological shifts and postoperative complications especially organ/space SSI after PD. Revealing PBD-associated microbiome alteration may facilitate optimization of perioperative antibiotic prophylaxis and decrease the occurrence of postoperative complications with consequent reduction on hospital length of stay and treatment cost.

## Methods

### Patients

This single-center retrospective study included patients undergoing PD between March 2018 and March 2024 at the Department of Pancreatic and Metabolic Surgery, Nanjing Drum Tower Hospital, Affiliated Hospital of Medical School, Nanjing University. The inclusion criteria were as follows: (a) patients who underwent PD, (b) no history of chemotherapy and/or radiotherapy, and (c) samples from preoperative and/or intraoperative bile cultures. In total, 758 patients underwent PD in our department between March 2018 and March 2024. The exclusion criteria were as follows: (a) simultaneous hepatic resection, colon resection, and/or total pancreatectomy; (b) emergency surgery for trauma; and (c) incomplete clinical data. The sample size (*n* = 323) was determined by retrospective availability of bile cultures from eligible PD patients treated during the study period (2018–2024). Of 756 patients initially screened, 433 patients were excluded: 413 due to unavailable bile cultures and 20 for incomplete medical records. The study was approved by the Health Research Ethics Board of the Drum Tower Hospital of Nanjing University Medical School (2024-795-01) and conducted in accordance with the Declaration of Helsinki. Written informed consent for all participants in this study has been obtained.

### Surgery and perioperative management

Pancreaticojejunostomy (PJ) was routinely performed *via* manual duct-to-mucosal, end-to-side, and double-layer interrupted anastomosis routinely [14]. Hepaticojejunostomy and gastrojejunostomy was performed by end-to-side anastomosis with monolayer continuous suture [15]. PBD was indicated for patients meeting one of the following criteria: serum total bilirubin level ≥15 mg/dl, preoperative cholangitis, or jaundice with malnutrition requiring nutritional support [16]. PBD was preferentially achieved using 10-Fr plastic stents *via* endoscopic nasobiliary drainage (ENBD), and percutaneous transhepatic choledochal drainage (PTCD) was applied for ENBD failure cases. Consistent with our prior protocol [5], drainage duration was limited to ≤2 weeks when clinically feasible.

The bile specimen obtained immediately after the bile duct transected was injected into blood culture vial of automated blood culture system (BACT/ALERT VIRTUO). When the system showed a positive growing signal, the sample was extracted from the bottle for Gram staining or Acid-fast staining to make smear microscopy, followed by subculture on Columbia blood agar and MaConkey Agar plate at 37 °C with 5% CO_2_. The pathogens cultured on the agar were further identified by matrix assisted laser desorption ionization time of flight (MALDI-TOF) mass spectrometry (VITEK MS system, BioMérieux, France). Antibiotic susceptibility testing was performed using the automated broth microdilution system (VITEK^®^2, bioMérieux) with AST-N335 and AST-XN04 card. Testing results were interpreted according to Clinical and Laboratory Standards Institute (CLSI) M-100, 34th edition recommended breakpoints [17].

Third-generation cephalosporins, such as ceftriaxone, were administered during surgery and extended for as long as 48 h as prophylactic antibiotics. The choice of prophylactic antibiotics was adjusted accordingly if bile culture results were available prior to surgery. Postoperative drainage fluid tests, including amylase and bacterial culture, were performed on POD 1, 3, and 5, and every 2–3 days thereafter until all drains were removed.

### Clinical data collection and definition of complication

Information regarding preoperative demographic data, intraoperative variables, and postoperative complications was collected from the institutional medical record system. SSI, which were divided into incisional and organ/space SSI, were diagnosed according to the Centers for Disease Control and Prevention (CDC) guidelines [3]. Postoperative complications were categorized using the Clavien–Dindo classification, with major complications defined as ≥ grade III. The definitions of CR-POPF, delayed gastric emptying (DGE), chyle leakage (CL), and post-pancreatectomy hemorrhage (PPH) were based on the International Study Group of Pancreatic Surgery (ISGPS) [18–21]. The diagnostic criteria for bile leakage (BL) were based on the guidelines of the International Study Group of Liver Surgery [22].

### Statistical analysis

All clinical data were analyzed using SPSS version 26.0 software for Windows (SPSS Inc.). Pearson’s *χ^2^* test or Fisher’s exact test was performed for categorical variables and presented as frequencies and percentages. Normally distributed continuous variables were analyzed using an independent *t* test, which was expressed as mean ± standard deviation (SD). Continuous variables that were distributed non-normally are shown as median (interquartile range, IQR) and were compared using the Mann–Whitney *U* test. Multivariable logistic regression model identified independent risk factors for organ/space SSI. Clinically relevant covariates (e.g. patient demographics, perioperative variables) and microorganisms with *p* < 0.10 on univariate analysis were initially considered. Variables were selected *via* backward LR method (entry probability 0.05, removal probability 0.10, maximum iterations 20). Multicollinearity was assessed using variance inflation factors (VIF <5 indicated acceptable collinearity). Model fit was confirmed *via* Hosmer–Lemeshow goodness-of-fit test. Odds ratios (OR) and 95% confidence intervals (95% CI) were calculated. Statistical significance was set at *p* < 0.05.

## Results

### Baseline characteristics

The cohort included 323 patients (median age of 62.5 years). 296 (91.6%) patients were treated by open technique and 27 (8.4%) patients were done by laparoscopic technique. 201 (62.2%) were diagnosed with preoperative jaundice and 94 (29.1%) with pancreatic ductal adenocarcinoma (PDAC). The other clinicopathological data are presented in Table 1. Patients were categorized into two groups depending on whether PBD was administered. A total of 191 (59.1%) patients were enrolled in the PBD group, and 132 (40.9%) patients were included in the non-PBD group. Statistical tests showed significant differences between preoperative jaundice, pathology, intraoperative blood loss, and blood transfusion volume (*p* < 0.05, Table 1). Organ/space SSI was more common in PBD group than in non-PBD group (*p* < 0.05, Table S1). There were no statistically significant differences in the rate of other common postoperative complications such as CR-POPF. Multivariate analysis identified that PBD (OR = 1.837, 95% CI: 1.158–2.916, *p* = 0.010) was one of the independent risk factors increasing the risk of organ/space SSI (Table S2).

**Table 1.** Patients characteristics.

Characteristics	All cohorts (*n* = 323)	PBD group (*n* = 191)	Non-PBD group (*n* = 132)	*p* value
Age (median, IQR), years	62.5 ± 11.1	63.4 ± 11.5	61.3 ± 10.6	0.106
BMI (mean ± SD), kg/m^2^	22.7 ± 2.9	22.5 ± 2.8	22.8 ± 2.9	0.350
Gender, *n* (%)				0.909
Male	197(61.0)	116(60.7)	81(61.4)	
Female	126(39.0)	75(39.3)	51(38.6)	
Hypertension, *n* (%)	120(37.2)	75(39.3)	45(37.5)	0.344
DM, *n* (%)	60(18.6)	37(19.4)	23(17.4)	0.658
Smoking, *n* (%)	93(28.8)	50(26.2)	43(32.6)	0.212
Drinking, *n* (%)	66(20.4)	37(19.4)	29(22.0)	0.569
Preoperative jaundice, *n* (%)	201(62.2)	163(85.3)	38(28.8)	<0.001
Pathology, *n* (%)				<0.001
PDAC	94(29.1)	52(27.2)	42(31.8)	
DCC	46(14.2)	139(72.8)	90(68.2)	
VAC	137(42.4)	97(50.8)	40(30.3)	
IPMN	10(3.1)	0(0.0)	10(7.6)	
SPN	3(0.9)	0(0.0)	3(2.3)	
SCN	4(1.2)	2(1.0)	2(1.5)	
Others	29(9.0)	6(3.1)	23(17.4)	
Type of resection				0.314
PD	138(42.7)	86(45.0)	52(39.4)	
PPPD	185(57.3)	105(55.0)	80(60.6)	
Surgical method				0.406
Laparoscopic	27(8.4)	18(9.4)	9(6.8)	
Open	296(91.6)	173(90.6)	123(93.2)	
Vessel resection, *n* (%)				0.953
Yes	29(9.0)	17(8.9)	12(9.1)	
No	294(91.0)	174(91.1)	120(90.9)	
Pancreatic consistency, *n* (%)				0.588
Hard	71(22.0)	40(20.9)	31(23.5)	
Soft	252(78.0)	151(79.1)	101(76.5)	
Diameter of MPD (median, IQR), mm	3.0(2.0–4.0)	3.0(2.0–4.0)	2.5(2.0–4.0)	0.737
Operation time (median, IQR), min	350.0(280.0–420.0)	340.0(280.0–410.0)	350.0(283.8–420.0)	0.543
Blood loss volume (median, IQR), ml	400.0(250.0–600.0)	400.0(300.0–600.0)	300.0(200.0–600.0)	0.032
Blood transfusion volume (median, IQR), ml	300.0(0.0–800.0)	337.5(0.0–900.0)	0.0(0.0–618.8)	0.032

IQR: interquartile range; SD: standard deviation; BMI: body mass index; DM: diabetes mellitus; PBD: preoperative biliary drainage; PDAC: pancreatic ductal adenocarcinoma; DCC: distal cholangiocarcinoma; VAC: Vater’s ampullary carcinoma; IPMN: intraductal papillary mucinous neoplasm; SPN: solid pseudopapillary neoplasm of the pancreas; SCN: pancreatic serous cystadenoma; PD: pancreaticoduodenectomy; PPPD: pylorus-preserving pancreaticoduodenectomy; MPD: main pancreatic duct.

### Assessment of pathogens in bile

The distribution of bacteria in bile is shown in Table 2. A total of 210 (65.0%) patients were colonized with bacteria and 96 (29.7%) patients were identified with polymicrobial mixed flora. Dominant species detected in bile included *K. pneumoniae* (14.2%), *E. coli* (11.1%), and *E. faecalis* (9.0%). Subgroup analysis demonstrated significantly higher rates of positive bile culture (90.6% vs 28.0%; *p* < 0.001) and polymicrobial colonization (47.1% vs. 4.5%; *p* < 0.001) (Table 2) in the PBD versus non-PBD group. Notably, *K. pneumoniae* was isolated in 23.6% of patients who underwent PBD compared to 0.8% of patients without PBD. Meanwhile, *E. faecalis* (14.1%), *E. faecium* (6.8%), *E. cloacae* (8.4%), *A. baumannii* (4.7%), and *Fungus* (4.2%) were predominant in patients with PBD.

**Table 2.** Microorganisms cultured from bile.

Microorganisms	All cohort (*n* = 323)	PBD group (*n* = 191)	Non-PBD group (*n* = 132)	*p* value
Positive, *n* (%)	210(65.0)	173(90.6)	37(28.0)	<0.001
Polymicrobial mixed flora, *n* (%)	96(29.7)	90(47.1)	6(4.5)	<0.001
*K. pneumoniae*, *n* (%)	46(14.2)	45(23.6)	1(0.8)	<0.001
*E. faecalis*, *n* (%)	29(9.0)	27(14.1)	2(1.5)	<0.001
*S. epidermidis*, *n* (%)	11(3.4)	9(4.7)	2(1.5)	0.210
*E. faecium*, *n* (%)	14(4.3)	13(6.8)	1(0.8)	0.009
*S. haemolyticus*, *n* (%)	5(1.5)	5(2.6)	0(0.0)	0.082
*E. coli*, *n* (%)	36(11.1)	25(13.1)	11(8.3)	0.182
*Fungus*, *n* (%)	8(2.5)	8(4.2)	0(0.0)	0.023
*E. cloacae*, *n* (%)	17(5.3)	16(8.4)	1(0.8)	0.002
*P. aeruginosa*, *n* (%)	6(1.9)	4(2.1)	2(1.5)	1.000
*A. baumannii*, *n* (%)	9(2.8)	9(4.7)	0(0.0)	0.012
*S. aureus*, *n* (%)	6(1.9)	6(3.1)	0(0.0)	0.085

PBD: preoperative biliary drainage.

### Resistant patterns to antibiotics

Table 3 summarizes the antibiotic resistance patterns according to the PBD. Resistance to piperacillin–tazobactam occurred in 23.1% of patients in the PBD group compared with 0.0% in the non-PBD group (*p* < 0.05). Similarly, resistance to cefoperazone–sulbactam, ciprofloxacin, and levofloxacin was significantly higher in the PBD group than in the non-PBD group (*p* < 0.05).

**Table 3.** Antibiotic resistance pattern according to the preoperative biliary drainage.

Resistance against antibiotics	PBD		*p* value
	Yes	No	
Piperacillin–tazobactam, *n* (%)	21 of 91(23.1)	0 of 16(0.0)	0.038
Cefoperazone–sulbactam, *n* (%)	23 of 91(25.3)	0 of 16(0.0)	0.021
Cefepime, *n* (%)	20 of 91(22.0)	1 of 16(6.3)	0.187
Ciprofloxacin, *n* (%)	41 of 97(36.1)	1 of 16(6.3)	0.006
Levofloxacin, *n* (%)	55 of 115(47.4)	1 of 21(4.8)	<0.001
Imipenem, *n* (%)	7 of 91(7.7)	0 of 16(0.0)	0.591
Penicillin, *n* (%)	31 of 85(36.5)	5 of 12(41.7)	0.757
Ceftriaxone, *n* (%)	1 of 38(0.9)	0 of 6(0.0)	1.000
Oxacillin, *n* (%)	14 of 21(66.7)	2 of 3(66.7)	1.000
Clindamycin, *n* (%)	8 of 15(53.3)	1 of 2(50.0)	0.929
Ampicillin, *n* (%)	5 of 36(13.9)	0 of 3(0.0)	0.489

PBD: preoperative biliary drainage.

Subgroup analyses targeting antibiotic resistance of bacteria in the bile based on the presence of preoperative cholangitis found that the antibiotic resistance of piperacillin–tazobactam (36.6% vs 9.1%, *p* < 0.05), cefoperazone–sulbactam (36.6% vs 12.1%, *p* < 0.05), cefepime (29.3% vs 13.6%, *p* < 0.05), ciprofloxacin (57.1% vs 25.5%, *p* < 0.05), levofloxacin (58.3% vs 31.8%, *p* < 0.05), and ampicillin (26.7% vs 4.2%, *p* < 0.05) were significantly higher in the PBD group (Table S3).

Preoperative antibiotic treatment was associated with a significant increase in antibiotic resistance to piperacillin–tazobactam (33.3% vs 13.3%, *p* < 0.05), cefoperazone–sulbactam (33.3% vs 14.7%, *p* < 0.05), ciprofloxacin (56.1% vs 26.4%, *p* < 0.05), and levofloxacin (59.2% vs 31.0%, *p* < 0.05) (Table S4).

### Impact of bacterobilia on patients’ outcomes

CR-POPF, major complications, and organ/space SSI occurred in 26.9%, 20.1%, and 46.1% of patients in the current study, respectively. To investigate the effect of bacterobilia on postoperative complications, we performed subgroup analyses based on the presence of positive bile cultures. Only the occurrence of organ/space SSI occurred more frequently in patients with positive bile cultures (53.3% vs 32.7%, *p* < 0.05). Nevertheless, there were no significant differences in PPH, BL, incisional SSI, DGE, or CL between the two groups (*p* > 0.05) (Table 4).

**Table 4.** Surgical outcome of according to the bacterial culture of lavage fluid.

Complications	All cohort (*n* = 323)	Positive bile culture (*n* = 210)	Negative bile culture (*n* = 113)	*p* value
Severe complications				
Pancreatic fistula, *n* (%)				0.091
Non-PF/biochemical fistula	236(71.4)	147(70.0)	89(78.8)	
CR-POPF	87(26.9)	63(30.0)	24(21.2)	
Major complication, *n* (%)	65(20.1)	48(22.9)	17(15.0)	0.095
PPH, *n* (%)	27(8.4)	18(8.6)	9(8.0)	0.851
BL, *n* (%)	19(5.9)	9(4.3)	10(8.3)	0.096
Organ/space SSI, *n* (%)	149(46.1)	112(53.3)	37(32.7)	<0.001
Non-severe complication				
Incisional SSI, *n* (%)	11(3.4)	10(4.8)	1(0.9)	0.105
DGE, *n* (%)	77(23.8)	56(26.7)	21(18.6)	0.104
CL, *n* (%)	70(21.7)	46(21.9)	24(21.2)	0.890

PF: pancreatic fistula; CR-POPF: Clinically relevant postoperative pancreatic fistula (Grade B/C); BL: biliary leakage; CL: chyle leakage; PPH: post-pancreatectomy hemorrhage; DGE: delayed gastric emptying; SSI: surgical site infection.

### Bacterobilia and organ/space SSI

Table 5 presents the relationship between the types of organisms isolated from bile and organ/space SSI. *K. pneumoniae* and *P. aeruginosa* identified by univariate analysis were included in the subsequent multivariate analysis. *K. pneumoniae* in the bile was an independent risk factor for organ/space SSI (OR = 2.636, 95% CI: 1.353–5.137, *p* = 0.004).

**Table 5.** Association between type of microorganism cultured from bile and organ/space surgical site infection.

Microorganisms	All cohort (*n* = 323)	Organ/space SSI (*n* = 149)	*p* value	Logistic regression analysis	
				OR (95%CI)	*p* value
*K. pneumoniae*, *n* (%)			0.002	2.785(1.438–5.393)	0.002
Positive	46(14.2)	31(20.8)			
Negative	277(85.8)	118(79.2)			
*E. faecalis*, *n* (%)			0.808		
Positive	29(9.0)	14(9.4)			
Negative	294(91.0)	135(90.6)			
*S. epidermidis*, *n* (%)			0.236		
Positive	11(3.4)	7(4.7)			
Negative	312(96.6)	142(95.3)			
*E. faecium*, *n* (%)			0.398		
Positive	14(4.3)	8(5.4)			
Negative	309(95.7)	141(94.6)			
*S. haemolyticus*, *n* (%)			0.126		
Positive	5(1.5)	4(2.7)			
Negative	318(98.5)	145(97.3)			
*E. coli*, *n* (%)			0.889		
Positive	36(11.1)	17(11.4)			
Negative	287(88.9)	132(88.6)			
*Fungus*, *n* (%)			0.097		
Positive	8(2.5)	6(4.0)			
Negative	315(97.5)	143(96.0)			
*E. cloacae*, *n* (%)			0.937		
Positive	17(5.3)	8(5.4)			
Negative	306(94.7)	141(94.6)			
*P. aeruginosa*, *n* (%)			0.065		
Positive	6(1.9)	5(3.4)			
Negative	317(98.1)	144(96.6)			
*A. baumannii*, *n* (%)			0.210		
Positive	9(2.8)	6(4.0)			
Negative	314(97.2)	143(96.0)			
*S. aureus*, *n* (%)			0.308		
Positive	6(1.9)	4(2.7)			
Negative	317(97.3)	145(97.3)			

OR: odds ratio; CI: confidence interval; SSI: surgical site infection.

## Discussion

The current retrospective study revealed that PBD was associated with bacterobilia, alterations in its composition and organ/space SSI, aligning with prior findings [3,8,23,24]. Meanwhile, bacterial resistance was higher in patients with PBD, which provides a rationale for the subsequent adjustment of perioperative prophylactic antibiotics or therapeutic antibiotics after PD.

PBD was originally designed as a ‘bridge therapy’ before radical resection to restore enterohepatic circulation of bile and impair hepatic function due to obstructive jaundice [25]. Nevertheless, there remains considerable controversy regarding the clinical impact of PBD in patients undergoing PD. In the present study, we observed that patients with PBD were more likely to have an increased volume of intraoperative blood loss, blood transfusion and organ/space SSI. A possible explanation could be that biliary catheterization could exacerbate inflammation and edema in the region where the operation is performed, making the procedure more difficult to perform.

Several studies [26–28] suggested that PBD appeared to promote biliary bacterial colonization and postoperative SSI in patients undergoing PD, a finding consistent with our previous study [5]. The destruction of sphincter function of the ampulla of Vater and intervention of the bile duct system may induce pathogen colonization and a shift in the hepatobiliary system. In terms of bile microbiology, the current study identified that bacterobilia was more frequently detected in patients with PBD. The most commonly detected microorganisms in bile were *K. pneumoniae* (14.2%), *E. coli* (11.1%), *E. faecalis* (9.0%), and *E. cloacae* (5.3%), which were predominant in normal intestinal flora and in line with bacterobilia profiles from other studies [29,30]. Scheufele et al. conducted a retrospective study that enrolled 290 patients and revealed that PBD significantly increased the rate of positive bile culture as well as the occurrence of *E. coli* and *E. faecalis* [31]. We found that PBD was associated with an increase in bacterobilia, polymicrobial mixed flora contamination in bile, and the detection rate of *K. pneumoniae*, *E. faecalis*, *E. faecium*, *S. haemolyticus*, *Fungus*, *E. cloacae* and *A. baumannii* which corresponded to the results of multiple studies [28,32]. Additionally, of the antibiotics tested, we showed that the resistance rates to classical antibiotics (e.g. piperacillin–tazobactam, cefoperazone–sulbactam, ciprofloxacin and levofloxacin) were significantly higher in the PBD group. This study focused on the impact of bacterobilia in patients receiving PD and revealed that PBD, preoperative cholangitis, and preoperative antibiotic treatment were related to increased resistance rates to several classic antibiotics [31]. Thus, catheter insertion should be performed in selected patients, as previously reported [5].

Few studies examined the impact of bacterobilia on perioperative complications following PD specifically, and the existing findings remain inconsistent and inconclusive [28,29,33]. In the current study, we showed a significantly increased incidence of postoperative organ/space SSI in patients with positive bile cultures (53.3% vs 32.7%, *p* < 0.05). This finding aligns with other studies showing an association between biliary bacteria and the infectious complications [26,29,34]. The study conducted by Parapini et al. analyzed the effect of bacterobilia on postoperative complications in 162 patients [35]. Bile contamination was detected in 136 (84%) patients, polymicrobial infection was detected in 100 (74%), and an increase in bacterobilia was identified in patients with PBD. Their study further revealed that patients with bacteremia had a significantly higher rate of SSI (*p* < 0.05), with *Klebsiella* and *Enterococcus* being the main bacteria. The high rate of CR-POPF, major complications, and organ/space SSI observed in our study may be attributable to the fact that bile samples were obtained from patients whose bile appeared to be infectious or from those who received PBD. Thus, the selection bias of relatively high-risk patients was responsible for the comparatively high incidence of postoperative complications.

While our study identified bile colonization with *K. pneumoniae* as one of risk factors for organ/space SSI, the absence of molecular typing to confirm strain identity between bile and SSI sites limits causal inference. Future studies using genomic sequencing are needed to establish whether SSI pathogens originate from biliary contamination. Our previous study demonstrated that the presence of *K. pneumoniae* in the postoperative abdominal drainage fluid was significantly associated with morbidities involving CR-POPF and organ/space SSI after PD [15]. The study conducted by Rogers et al. investigated the microbiomes of pancreatic juice, bile, jejunal fluid, and feces from patients receiving PD using 16S sequencing and found similar microbial communities in all samples [36]. In another study, the similarity of pathogens in both bile and surgical sites was described [3]. The above findings suggest that bacteria colonizing the bile may translocate into the abdomen and all anastomoses that induce postoperative morbidities.

The findings of current study highlighted the importance for surgeons to consider the presence of bacterobilia. This carried significant implications for both the application of perioperative antibiotics and the potential need for early adjustment to targeted antibiotics therapy guided by drug susceptibility results, in managing postoperative infectious complications. It should be noted, however, that the heterogeneity in the types and antibiotic resistance patterns of pathogens isolated from bile across different institutions merits consideration. Antibiotic regimens providing appropriate coverage may need refinement based on local microbiological profiles. Additionally, for patients with positive bile cultures, we suggest vigilant postoperative monitoring of clinical signs and laboratory indicators. Should signs of infections (such as fever or elevated inflammatory markers) arise in the early postoperative period, antibiotic therapy should be adjusted promptly based on the culture results.

The current study had several limitations. First, it was a single-center retrospective study, which inherent selection bias existed such as higher rate of preoperative jaundice (85.3% vs. 28.8%). Although we adjusted for these confounders in multivariate regression models, residual confounding remains plausible. Second, notably, perioperative management protocols (e.g., antibiotic regimens, drain management duration/material) vary substantially across institutions, which may impact generalizability. At our center, standardized protocols minimized such heterogeneity-all PDs followed uniform antibiotic prophylaxis (ceftriaxone) and drainage duration (≤2 weeks) criteria when clinically feasible. Critically, biliary microbiome profiles and resistance patterns may exhibit geographic and institutional variations. Therefore, further multicenter and randomized controlled trials are essential to confirm the significance of bacterobilia in postoperative morbidities. Finally, there is a lack of molecular evidences linking *K. pneumoniae* in bile to SSI pathogens.

## Conclusions

In conclusion, the outcomes of our study showed that a positive bile culture was significantly associated with postoperative organ/space SSI after PD. Furthermore, PBD was associated with altered biliary microbiome composition, increased antibiotic resistance, and higher rates of organ/space SSI. At the same time, the incidence of organ/space SSI was significantly higher in patients with positive *K. pneumoniae* detection from bile. Thus, preoperative bile culture which help alter targeted subsequent antibiotic therapy during the perioperative course may be warranted, though further studies are needed to confirm causality between bile and SSI pathogens.

## Ethics approval and consent to participate section

This study was approved by the Health Research Ethics Board of the Drum Tower Hospital of Nanjing University Medical School (2024-795-01). Written informed consent for all participants was obtained from all subjects and/or their legal guardians (s).

## Consent for publication

Written informed consent for publication was obtained from all participants.

## Supplementary Material

Clean copy - Supplemental_table - IANN-2025-0156.R1.docx

## Data Availability

The datasets generated and/or analyzed during the present study are not publicly available because of patient privacy concerns but are available from the corresponding author (Xu Fu, fuxunju2012@163.com) upon reasonable request.
