# Supplementary material for: The impact of preoperative biliary drainage on bile colonization of patients undergoing pancreaticoduodenectomy
Source: Ann Med. 2025 Jul 31;57(1):2540024. doi: 10.1080/07853890.2025.2540024 (PMC12315152; doi:10.1080/07853890.2025.2540024)
Supplement: Clean copy - Supplemental_table - IANN-2025-0156.R1.docx [file IANN_A_2540024_SM4473.docx]

*Table S1 Surgical outcomes according to preoperative biliary drainage*

| Complications | All cohort (n=323) | PBD group (n=191) | Non-PBD group (n=132) | *P* value |
| --- | --- | --- | --- | --- |
| Severe complications |  |  |  |  |
| Pancreatic fistula, n (%) |  |  |  | 0.692 |
| non-PF/biochemical fistula | 236(71.4) | 138(72.3) | 98(74.2) |  |
| CR-POPF | 87(26.9) | 53(27.7) | 34(25.8) |  |
| Major complication, n (%) | 65(20.1) | 42(22.0) | 23(17.4) | 0.314 |
| PPH, n (%) | 27(8.4) | 17(8.6) | 10(7.6) | 0.672 |
| BL, n (%) | 19(5.9) | 10(7.6) | 9(4.7) | 0.282 |
| Organ/space SSI, n (%) | 149(46.1) | 99(51.8) | 50(37.9) | 0.013 |
| Non-severe complication |  |  |  |  |
| Incisional SSI, n (%) | 11(3.4) | 7(3.7) | 4(3.0) | 0.757 |
| DGE, n (%) | 77(23.8) | 51(26.7) | 26(19.7) | 0.146 |
| CL, n (%) | 70(21.7) | 42(22.0) | 28(21.2) | 0.868 |

*PF: pancreatic fistula; CR-POPF: Clinically relevant postoperative pancreatic fistula (Grade B/ C); BL: biliary leakage; CL: chyle leakage; PPH: post-pancreatectomy hemorrhage; DGE: delayed gastric emptying; SSI: surgical site infection*

*Table S2 Univariate and multivariate analysis of organ/space surgical site infection*

| Variables | Univariate analysis | | Multivariate analysis | |
| --- | --- | --- | --- | --- |
|  | OR (95%CI) | *P* value | OR (95%CI) | *P* value |
| Age | 1.006(0.986-1.026) | 0.571 |  |  |
| Body mass index | 1.107(1.024-1.198) | 0.011 | 1.097(1.011-1.190) | 0.027 |
| Gender | 1.061(0.677-1.661) | 0.797 |  |  |
| Diabetes mellitus | 1.249(0.708-2.203) | 0.443 |  |  |
| Hypertension | 1.092(0.694-1.716) | 0.704 |  |  |
| Preoperative Jaundice | 1.398(0.887-2.202) | 0.149 |  |  |
| Preoperative biliary drainage | 1.765(1.123-2.773) | 0.014 | 1.837(1.158-2.916) | 0.010 |
| Pathology diagnosis | 1.569(0.962-2.562) | 0.071 |  |  |
| Surgical method | 1.077(0.487-2.380) | 0.854 |  |  |
| Vessel resection | 0.782(0.364-1.678) | 0.527 |  |  |
| Pancreas consistency | 0.880(0.518-1.496) | 0.637 |  |  |
| Diameter of MPD | 0.961(0.846-1.092) | 0.546 |  |  |
| Operating time | 1.003(1.001-1.005) | 0.014 | 1.002(1.000-1.005) | 0.060 |
| Blood loss volume | 1.000(1.000-1.001) | 0.435 |  |  |
| Blood transfusion | 1.000(1.000-1.001) | 0.174 |  |  |

*MPD: main pancreatic duct*

*Table S3 Antibiotic resistance pattern according to the presence of cholangitis*

| Resistance against antibiotic | Preoperative cholangitis | | *P* value |
| --- | --- | --- | --- |
|  | Yes | No |  |
| Piperacillin-tazobactam, n (%) | 15 of 41(36.6) | 6 of 66(9.1) | <0.001 |
| Cefoperazone-sulbactam, n (%) | 15 of 41(36.6) | 8 of 66(12.1) | 0.003 |
| *Cefepime*, n (%) | 12 of 41(29.3) | 9 of 66(13.6) | 0.048 |
| *Ciprofloxacin*, n (%) | 24 of 42(57.1) | 18 of 71(25.4) | 0.001 |
| *Levofloxacin* n (%) | 28 of 48(58.3) | 28 of 88(31.8) | 0.003 |
| *Imipenem*, n (%) | 3 of 41(7.3) | 4 of 66(6.1) | 1.000 |
| *Penicillin*, n (%) | 11 of 27(40.7) | 25 of 70 (35.7) | 0.646 |
| *Ceftriaxone*, n (%) | 0 of 11(0.0) | 1 of 33(3.0) | 1.000 |
| *Oxacillin*, n (%) | 14 of 21(66.7) | 2 of 3(66.7) | 1.000 |
| *Clindamycin*, n (%) | 1 of 3(33.3) | 8 of 14(57.1) | 0.576 |
| Ampicillin, n (%) | 4 of 15(26.7) | 1 of 24(4.2) | 0.062 |

*Table S4* *Antibiotic resistance pattern according to the preoperative antibiotic treatment*

| Resistance against antibiotic | Preoperative antibiotics | | P value |
| --- | --- | --- | --- |
|  | Yes | No |  |
| Piperacillin-tazobactam, n (%) | 13 of 39(33.3) | 8 of 68(13.3) | 0.007 |
| Cefoperazone-sulbactam, n (%) | 13 of 39(33.3) | 10 of 68(14.7) | 0.024 |
| *Cefepime*, n (%) | 10 of 39(25.6) | 11 of 68(16.2) | 0.235 |
| *Ciprofloxacin*, n (%) | 23 of 41(56.1) | 19 of 72(26.4) | 0.002 |
| *Levofloxacin* n (%) | 29 of 49(59.2) | 27 of 87(31.0) | 0.001 |
| *Imipenem*, n (%) | 3 of 39(7.7) | 4 of 68(5.9) | 0.704 |
| *Penicillin*, n (%) | 13 of 29(44.8) | 23 of 68(33.8) | 0.304 |
| *Ceftriaxone*, n (%) | 0 of 11 (0.0) | 1 of 33(3.0) | 1.000 |
| *Oxacillin*, n (%) | 5 of 8(62.5) | 11 of 16(68.8) | 1.000 |
| *Clindamycin*, n (%) | 2 of 7 (28.6) | 7 of 10(70.0) | 0.153 |
| Ampicillin, n (%) | 3 of 16(18.8) | 2 of 23(8.7) | 0.631 |
